# UNVEILING TENSIONS: CAREGIVING FOR OLDER ADULTS WITH CANCER IN THE COVID-19 ERA

**DOI:** 10.1093/geroni/igad104.3683

**Published:** 2023-12-21

**Authors:** Charlotte Weiss, Rachel Johnson-Koenke, Karen Sousa, Connie Ulrich, Karen Hirschman

**Affiliations:** University of Pennsylvania, Philadelphia, Pennsylvania, United States; University of Colorado- Anschutz Medical Campus, Aurora, Colorado, United States; University of Colorado- Anschutz Medical Campus, Aurora, Colorado, United States; University of Pennsylvania, Philadelphia, Pennsylvania, United States; University of Pennsylvania, Philadelphia, Pennsylvania, United States

## Abstract

The COVID-19 pandemic was traumatic for many individuals, including older adults with serious illness and their families. This qualitative study aimed to explore family caregiver narratives for the historical, societal, and institutional context of caring for older adults with advanced cancer during the COVID-19 pandemic. Using narrative inquiry alongside photo elicitation, 5 caregiver narratives were co-created during in-person semi-structured interviews or virtually through Zoom video conferencing. Each of the participant caregivers started caring for an older adult family member with cancer during the COVID-19 pandemic. Narratively derived thematic threads of COVID-19 emerged as short stories of tension woven throughout the cancer caregiver narratives. The themes within and across the narratives included: advocacy of quality healthcare for care recipients, protection of care recipients from COVID-19; protection of care recipients from medical institutional disregard; financial strain and uncertainty; lack of medical, emotional, social, and spiritual support; intention towards self-care; and saying goodbye in isolation. The backdrop of COVID-19 contributed to caregiver fear, anxiety, feelings of being alone, guilt, and emotional and physical exhaustion. Findings from this study highlight that cancer family caregivers experienced multiple layers of tension when caring for older adults with advanced cancer during COVID-19. By acknowledging tensions emergent through storytelling, researchers and clinicians contribute to valuing the diversity of individually and collectively lived human caregiving experiences and the uncertainty and turmoil in their lives.

